# Shoulder‐Tip Pain After Laparoscopic Surgery: A Narrative Review of Intraperitoneal Anaesthesia

**DOI:** 10.1111/ans.70746

**Published:** 2026-05-19

**Authors:** Jason Arsanious, Alice Osterwalder, Sarah K. Thompson

**Affiliations:** ^1^ College of Medicine & Public Health Flinders University Bedford Park South Australia Australia; ^2^ Discipline of Surgery Flinders Medical Centre Bedford Park South Australia Australia; ^3^ Adelaide Gastrointestinal Specialists Eastwood South Australia Australia

**Keywords:** insufflation pressure, intraperitoneal anaesthetic, laparoscopic surgery, minimally invasive surgery, postoperative pain, shoulder‐tip pain

## Abstract

**Background and Aims:**

Shoulder‐tip pain is a common and distressing side effect following laparoscopic surgery. Standard systemic analgesic regimens are used to manage postoperative pain but are often associated with side effects such as nausea and vomiting. There is some evidence to support a reduction in insufflation pressure to lessen shoulder‐tip pain. This narrative review examined the efficacy of intraperitoneal anaesthesia on shoulder‐tip and overall pain after laparoscopic surgery.

**Methods:**

Relevant literature was identified through database searches containing terms related to intraperitoneal anaesthesia, insufflation pressure, laparoscopic surgery, shoulder‐tip pain and postoperative pain.

**Results:**

Most studies evaluating intraperitoneal local anaesthesia used bupivacaine or ropivacaine. There was a wide variety of surgical procedures with limited reporting of shoulder‐tip pain. Mixed results were found regarding the efficacy of intraperitoneal anaesthesia on total pain scores.

**Conclusions:**

Intraperitoneal anaesthesia may be an effective strategy to reduce postoperative shoulder‐tip and overall pain scores following laparoscopic surgery, but the evidence remains inconclusive due to methodological heterogeneity. Further well‐designed studies, particularly in upper gastrointestinal laparoscopic surgery, using standardised dosing of intraperitoneal anaesthesia, standard versus low insufflation pressure, and validated outcome measures are required to determine its clinical efficacy.

## 1 Introduction

Minimally invasive surgery has become the gold standard for many procedures as innumerable studies have shown its association with reduced postoperative pain, a shorter hospital stays and a faster recovery compared to open surgery [1]. Despite these benefits, post‐laparoscopic pain is frequently reported by patients. Types of pain include somatic pain from incisions and trocar sites, visceral pain from manipulation of internal organs, and referred pain, commonly shoulder‐tip pain [2, 3].

Shoulder‐tip pain is caused by irritation of the phrenic nerves, which originate from the anterior rami of C3–C5 and course through the thorax to pierce and innervate the diaphragm. Due to shared spinal roots with the supraclavicular nerves (C3–C4), phrenic nerve irritation during laparoscopic surgery may cause referred pain to the C4 dermatome, typically felt at the shoulder‐tip (Figure 1) [4, 5, 6, 7]. Phrenic nerve irritation is theorised to result from insufflation of the abdominal cavity and gas or fluid trapping beneath the diaphragm [3, 8, 9]. In upper gastrointestinal surgery, an additional mechanism may involve the introduction of sutures into the diaphragm, with localised injury to diaphragmatic phrenic nerve fibres.

**FIGURE 1:**
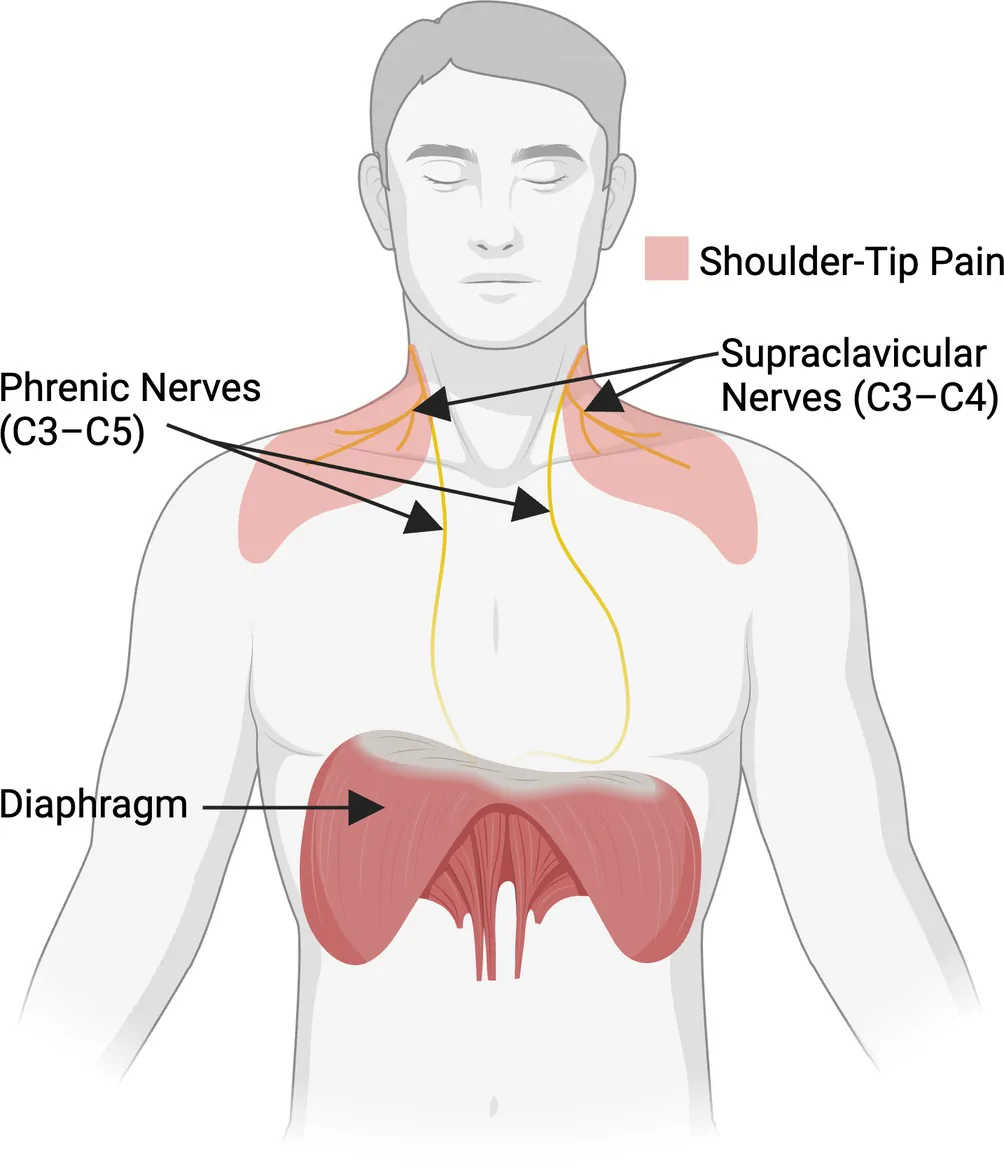
Referred diaphragmatic pain via the phrenic and supraclavicular nerves, presenting as shoulder‐tip pain (Created in BioRender (2026). https://BioRender.com/hiarjkl).

The occurrence of pain decreases patient satisfaction and often limits early ambulation and subsequent hospital discharge, increasing procedural costs [10]. Prevention of postoperative pain is essential for accelerating patient recovery and is integral to several Enhanced Recovery After Surgery (ERAS) programs. This review examines the literature on intraperitoneal anaesthesia in the management of shoulder‐tip pain in laparoscopic surgery.

## 2 Management of Shoulder‐Tip Pain

### 2.1 Opioid and Non‐Opioid Regimens

Parenteral opioids and non‐steroidal anti‐inflammatory drugs (NSAIDs) are the mainstay of postoperative pain relief [11]. Paracetamol, NSAIDs (intravenous or oral), COX‐2 inhibitors (e.g., parecoxib, diclofenac and dexketoprofen), dexamethasone, ropivacaine wound infiltration, and oxycodone have each been shown to significantly reduce postoperative pain [12, 13]. Whilst effective, these parenteral medications often cause side effects such as nausea and vomiting, which are best avoided in patients undergoing laparoscopic surgery [14]. Alternative treatments such as lidocaine patches have demonstrated efficacy in pain management; however, nausea remains a common side effect [15]. In line with Enhanced Recovery After Surgery (ERAS) protocols, multimodal analgesia and regional anaesthetic techniques, including interscalene and suprascapular nerve blocks, are increasingly being incorporated to enhance pain control whilst limiting opioid requirements [16].

### 2.2 Intra‐Abdominal Insufflation Pressure

Studies exploring the relationship between abdominal insufflation pressure and postoperative pain date back as far as 1997 [17]. Low insufflation pressure is defined as less than 10 mmHg, with an acceptable range of 7–9 mmHg. Standard insufflation pressures typically range from 12–15 mmHg, but adjustments may be required due to patient comorbidities and the extent of deep neuromuscular blockade [18]. It is important to note that many authors caution against low insufflation pressure during trocar insertion to avoid injury to underlying organs and vessels. Moreover, some investigators theorise that pre‐stretching of the abdominal wall musculature with a short period of standard insufflation pressure may increase the surgeon's working space during subsequent low insufflation pressure [19].

Many of the studies included in the latest systematic review and meta‐analysis are prospective, randomised controlled trials [19]. However, not all looked at pain as a primary outcome and the authors included urological procedures (including robotic prostatectomy). We have summarised relevant general surgical procedures in Table 1 [20, 21, 22, 23, 24, 25, 26, 27, 28, 29, 30, 31, 32, 33, 34]. Roughly half of the studies included found a significant reduction in postoperative pain, although not all studies looked specifically at shoulder‐tip pain.

**TABLE 1:** Prospective randomised controlled studies which examined the impact of low‐pressure carbon dioxide insufflation on postoperative pain in minimally invasive surgery.

Type of laparoscopic surgery	Study reference	Number of patients	Insufflation pressure (mmHg)	Significant shoulder‐tip pain reduction	Total pain effect	Other pros/cons
Cholecystectomy	[20]	148	7 versus 12	Yes	Reduced total pain and post‐operative analgesia	Improved quality of life; required reverse Trendelenburg positioning to improve abdominal exposure
	[21]	40	7 versus 12	No	No difference	Small sample size; feasible outpatient day surgery; limited follow up period; occasionally required increased pressures due to adhesions around Calot triangle
	[22]	140	7 versus 14	No	Similar pain scores and analgesic use	Surgery was feasible in 97% of low‐pressure cases; 2 patients required increased pressure due to obesity
	[23]	100	7–8 versus 12–13	Yes	Reduced total pain and post‐operative analgesia	Comparable intraoperative complication rate; administration of rescue analgesia may mask insufflation pressure effect
	[24]	60	8 versus 12 versus 14	No	No reduction in total pain	Female patients only; small sample size; increased operative duration at higher pressure (14 mmHg)
	[25]	26	8 versus 12	N/A	Reduced total pain and post‐operative analgesia	Small sample size; decreased hospital stay; resulted in better pulmonary function
	[26]	100	8 versus 14	Yes	N/A	Comparable operative duration; improved early quality of life
	[27]	90	9 versus 13	Yes	Reduced total pain at 12 h and 24 h and reduced post‐operative analgesia	No change in operative duration; increased technical difficulty at 9 mmHg
	[28]	109	10 versus 10 + saline versus 14	Yes	Reduced total pain and post‐operative analgesia in 10 mmHg groups	Saline improved pain; slightly increased operative time at low‐pressures
	[29]	50	10 versus 15	N/A	No difference	Small sample size; no effect on visceral/incisional pain
Gynaecological laparoscopy (except hysterectomy)	[30]	60	7 versus 15	Yes	Reduced pain scores at 4 h, 8 h and 24 h post‐operation	Small sample size; decreased end‐tidal CO_2_ and peak airway pressure; limited follow up period
	[31]	360	8 versus 15	No	No difference	A range of insufflation pressures investigated; very small clinical effect
	[32]	150	8 versus 12 versus 15	Yes	Reduced pain scores at 12 h and 24 h post‐operation; no difference in analgesic requirement	Lower pressure surgeries had significantly higher blood loss and longer operative time
Hysterectomy	[33]	42	8 versus 12	Yes	No difference	Small sample size
	[34]	178	8 versus 15	Yes	Reduced total pain and post‐operative analgesia	Decreased pCO2; decreased hospital stay; no increase in complications

Abbreviation: N/A = not assessed.

### 2.3 Intraperitoneal Local Anaesthesia

Intraperitoneal anaesthetic as a method of addressing shoulder‐tip pain after laparoscopy was first reported in the Lancet in 1991 [35]. Numerous local anaesthetic agents are available globally, with bupivacaine and ropivacaine as the most widely used for intraperitoneal anaesthesia (Table 2) [36, 37, 38, 39, 40, 41].

**TABLE 2:** Globally available and applicable local anaesthetic drugs, their maximum single dose and availability in Australia.

Anaesthetic agent	References	Maximum single dose (mg/Kg)	Available In Australia
Articaine	[36]	7	No
Bupivacaine	[37]	2	Yes
Chloroprocaine	[38]	11	No
Cocaine	[39]	1.5	Yes
Etidocaine	[40]	4.5	No
Levobupivacaine	[41]	2	No
Lidocaine (Lignocaine)	[37]	3	Yes
Mepivacaine	[41]	4.5	No
Prilocaine	[37]	6	Yes
Procaine	[41]	7	No
Ropivacaine	[37]	3	Yes
Sameridine		No available data	No
Tetracaine	[41]	1	Yes
Tonicaine		No available data	No

Table 3 summarises the current literature on the use of intraperitoneal anaesthesia in laparoscopic surgery [35, 42, 43, 44, 45, 46, 47, 48, 49, 50, 51, 52, 53, 54, 55, 56, 57, 58, 59, 60, 61, 62, 63, 64, 65, 66]. There was a wide variety in the surgical procedures performed, with high heterogeneity amongst studies with the choice of local anaesthetic agent, dose, timing and technique of agent administration, and timing and choice of outcome measurements. Surprisingly, many studies did not include separate data acquisition for shoulder‐tip pain. Once again, there were mixed results, with some studies showing a significant decrease in total pain and others showing no difference.

**TABLE 3:** Studies which looked at the impact of different intraperitoneal anaesthetics and doses on pain in minimally invasive surgery.

Anaesthetic agent	Study reference	Number of patients	Procedure	Dose	Significant shoulder‐tip pain reduction	Total pain effect	Other pros/cons
Bupivacaine	[23]	50	Roux‐en‐Y gastric bypass	50 mg	Yes	Reduced total pain without reduction in post‐operative analgesia	Short follow‐up with scheduled discharge within 24 h
	[24]	105	Cholecystectomy or fundoplication or appendicectomy or hernia repair or diagnostic laparoscopy	50 mg	Yes	Reduced total pain and post‐operative analgesia	No change in length of hospital stay; no evidence of Bupivacaine toxicity
	[25]	206	Cholecystectomy	50 mg	No	Not compared to placebo; unknown effect	Wound‐site bupivacaine may mask intraperitoneal analgesic effects in trials
	[26]	80	Gynaecology	50 mg	N/A	Significant total pain reduction	Small sample; short follow‐up
	[27]	120	Sleeve gastrectomy	75 mg	N/A	Reduced total pain	Reduction in post‐operative nausea and vomiting
	[28]	133	Roux‐en‐Y gastric bypass	75 mg	N/A	No reduction in total pain; Reduction in oral analgesic consumption	No difference in length of stay, intravenous analgesic, respiratory recovery and antiemetic use
	[29]	160	Hysterectomy	100 mg	No	No reduction in total pain	Short follow‐up; patient‐controlled anaesthetic may have masked intraperitoneal analgesic effects
	[30]	68	Sleeve gastrectomy	100 mg	No	Reduced total pain	Short follow‐up; results suggest patient sex may influence analgesic effects as only males reported total pain reduction
	[31]	60	Gynaecology	100 mg	No	Reduced total pain and post‐operative analgesia	Short follow‐up; time to first analgesia was significantly increased compared to placebo; bupivacaine was less effective at pain reduction than ropivacaine
	[32]	72	Gynaecology	100 mg	No	No reduction in total pain	Short follow‐up; extensive post‐operative multimodal analgesia may mask effect
	[33]	100	Bariatric	100 mg	No	Reduced total pain and post‐operative analgesia	No effect on nausea or vomiting
	[34]	106	Bariatric	100 mg	No	Reduced total pain and post‐operative analgesia	All patients received continuous intravenous paracetamol for 24 h postop
	[35]	79	Gynaecology	150 mg	No	Reduced total pain when given in pre‐ and postoperative regimens	Timing of anaesthetic and epinephrine co‐administration influenced outcomes
	[36]	120	Gynaecology	200 mg	N/A	Reduced total pain and post‐operative analgesia	Pre‐ and postoperative anaesthetic administration was tested but effect of timing unclear
	[37]	30	Laparoscopy	360 mg (continuous infusion over 48 h)	No	Reduced pain but no difference in analgesic use	Continuous infusion reduced pain but did not change analgesic consumption
	[37]	130	Adjustable gastric banding	500 mg	No	No reduction in total pain	High dose in obese patients showed no benefit
Bupivacaine + Epinephrine	[35]	79	Gynaecology	50 mg	No	No reduction in pain	Postoperative‐only dosing is less effective than a combination of pre‐ and postoperative dosing
	[35]	79	Gynaecology	150 mg	No	Reduced visceral pain	Trial delivered solution pre‐ and postoperatively
Bupivacaine + Magnesium Sulphate	[38]	20	Hysterectomy	200 mg bupivacaine + mgso4 (40 mL)	N/A	Significant pain reduction in the first 24 h	Combination therapy improved early pain but not at 7 days; small sample; rescue analgesia may have confounded results
Bupivacaine + Morphine	[39]	90	Gynaecology	75 mg + 2 mg	N/A	Reduced total pain and post‐operative analgesia	Morphine adjunct improved analgesic effect of bupivacaine
	[32]	72	Gynaecology	100 mg + 3 mg	No	No significant pain reduction or analgesic requirement	Small sample; multimodal analgesia may have disguised differences
Lidocaine (Lignocaine)	[25]	206	Cholecystectomy	200 mg	No	No significant pain reduction compared to bupivacaine	No analgesic benefit compared to bupivacaine; trial lacked placebo
	[40]	133	Fundoplication or hernia repair	250 mg (pre‐operative)	Yes	Reduced total pain and post‐operative analgesia	Pre‐operative dosing reduced pain after fundoplication
	[40]	133	Fundoplication or hernia repair	250 mg (post‐operative)	No	No significant reduction in total pain or analgesic usage	Analgesic timing mattered; postoperative dosing was ineffective
	[41]	80	Diagnostic laparoscopy	400 mg (80 mL 0.5% with adrenaline)	Yes	Significant total pain reduction	Trial had a long follow‐up time; required large lignocaine dose
Morphine (intraperitoneal)	[39]	90	Gynaecology	2 mg (adjunct with bupivacaine)	N/A	Significant total pain reduction	Intraperitoneal morphine adjunct enhanced analgesic effect over bupivacaine alone and a placebo
	[32]	72	Gynaecology	3 mg	No	No significant reduction in total pain or analgesic requirement	Small sample size and multimodal analgesia may have masked effects
Ropivacaine	[42]	87	Fundoplication or cholecystectomy	50 mg	No	Reduced total pain	No change in patient length of stay, analgesia use, or adverse events
	[43]	110	Sleeve gastrectomy	70 mg	N/A	Reduced total pain and post‐operative analgesia	Enhanced respiratory recovery and mobilisation; no change to nausea and vomiting
	[44]	48	Sleeve gastrectomy	100 mg	N/A	Reduced total pain and post‐operative analgesia	Potential improvement in efficacy when administered with dexmedetomidine
	[31]	60	Gynaecology	150 mg	No	Reduced total pain and post‐operative analgesia	Short follow‐up; time to first analgesia was significantly increased compared to placebo; Ropivacaine was more effective at pain reduction than Bupivacaine
	[45]	220	Laparoscopy	150 mg	No	No significant reduction in total pain or analgesic requirement	No difference in time to first ambulation, ingestion of food, severity of nausea and vomiting, duration of hospital stay, or satisfaction score
	[46]	92	Bariatric	200 mg	N/A	No significant reduction in total pain or analgesic requirement	Anaesthetic effect may have been masked by routine analgesic protocol
	[47]	110	Bariatric	300 mg	N/A	Reduced total pain and post‐operative analgesia	Ability for fluid intake increased; earlier mobilisation achieved; no change in nausea or vomiting
	[48]	104	Bariatric	Weight‐based dosing	N/A	Significant total pain reduction	Effect size variable; dosing reported per body weight; methodology unique to patient attributes
Tenoxicam	[26]	80	Gynaecology	20 mg	N/A	Significant total pain reduction	Small sample; short follow‐up
Tramadol	[26]	80	Gynaecology	50 mg	N/A	Significant total pain reduction	Small sample; short follow‐up

Abbreviation: N/A = not assessed.

## 3 Discussion

Shoulder‐tip pain is a common side effect associated with laparoscopic surgery and is caused by irritation to the phrenic nerves. Current management typically includes multimodal use of opioid and non‐opioid analgesia. Unfortunately, these systemic drugs often cause unwanted side effects, delay mobilisation, and result in longer hospital stays and decreased patient satisfaction.

Intra‐abdominal insufflation pressure may contribute to the intensity and duration of shoulder‐tip pain and overall pain scores, and has been well studied in patients undergoing laparoscopic cholecystectomy and gynaecological surgery. There was a paucity of studies in upper gastrointestinal surgery (laparoscopic fundoplication and/or hiatus hernia repair) and bariatric surgery. A recent systematic review and meta‐analysis reported compelling evidence from the inclusion of 85 studies (albeit, a wide range of surgical procedures including robotic prostatectomy) to support the use of lower insufflation pressure to reduce postoperative pain scores, the incidence of nausea and vomiting, and length of hospital stay [19].

Despite numerous randomised controlled trials, the current evidence does not identify an optimal intraperitoneal anaesthetic strategy. Varying drug choices, doses, timing of administration, technique of administration, and assessed outcomes impair the ability to make valid generalised conclusions. Many studies were underpowered and focused on small, single‐centre populations. A limited number of studies investigated shoulder‐tip pain [35, 42, 43, 44, 48, 49, 50, 51, 52, 53, 54, 56, 59, 60, 63], and comparisons amongst them revealed inconsistent results.

A dose–response relationship for any drug used as intraperitoneal anaesthetic has not been identified. Many trials stopped assessing patient pain within 24 h, so the impact of intraperitoneal anaesthetic on hospital length of stay, long‐term recovery and overall operative satisfaction remains unknown. All trials administered postoperative multimodal analgesia to address patient pain and, whilst this is ethical and necessary, it limited the attainable information about the efficacy of intraperitoneal anaesthesia. Sparse information was provided about the total length of stay for patients receiving intraperitoneal anaesthetic compared to placebo, and the economic viability of using intraperitoneal anaesthetic remains unknown.

## 4 Conclusion

Intraperitoneal anaesthetic offers an innovative approach to prevent and overcome postoperative pain; however, the evidence for intraperitoneal local anaesthesia remains inconsistent and requires further inquiry. It is also interesting to note that insufflation pressures may also influence postoperative shoulder‐tip pain yet most randomised trials did not specify the insufflation pressures used in their protocol. Based on our narrative review, further well‐designed randomised controlled trials are needed. Intraperitoneal local anaesthesia is a relatively inexpensive, widely applicable and easily implementable intervention to reduce shoulder‐tip pain after laparoscopic surgery and, as such, it warrants further high‐quality investigation.

## Funding

The authors have nothing to report.

## Disclosure

A/Prof Thompson is an Editorial Board member of *ANZ Journal of Surgery* and a co‐author of this article. To minimise bias, she was excluded from all editorial decision‐making related to the acceptance of this article for publication.

## Conflicts of Interest

The authors declare no conflicts of interest.

## Data Availability

Data sharing not applicable to this article as no datasets were generated or analysed during the current study.
